# Neuropsychological performance of Finnish and Egyptian children with autism spectrum disorder

**DOI:** 10.3402/ijch.v75.29681

**Published:** 2016-01-29

**Authors:** Sherin Elsheikh, Sanna Kuusikko-Gauffin, Marja-Leena Mattila, Katja Jussila, Hanna Ebeling, Soile Loukusa, Manal Omar, Geylan Riad, Arja Rautio, Irma Moilanen

**Affiliations:** 1 PEDEGO Research Unit, Department of Child Psychiatry, University of Oulu, Oulu, Finland; 2 Child Psychiatry Unit, Abbassia Mental Hospital, Cairo, Egypt; 3 Centre for Arctic Medicine, Thule Institute, University of Oulu, Oulu, Finland; 4 Clinic of Child Psychiatry, Oulu University Hospital, Oulu, Finland; 5 Faculty of Humanities, Logopedics, Child Language Research Centre, University of Oulu, Oulu, Finland; 6 Institute of Postgraduate Childhood Studies, Ain Shams University, Cairo, Egypt; 7 Faculty of Arts, Department of Psychology, Helwan University, Cairo, Egypt

**Keywords:** autism spectrum disorder, NEPSY, neuropsychology, neuropsychological abilities, culture

## Abstract

**Background:**

Previous studies investigating neuropsychological functioning of children with autism spectrum disorder (ASD) have only analysed certain abilities, such as executive functions or language. While comprehensive assessment of the neuropsychological profile of children with ASD has been the focus of recent research, most of the published evidence originates from single centres. Though studies on differences in neuropsychological features of children with ASD across countries are essential for identifying different phenotypes of ASD, such studies have not been conducted.

**Objective:**

Our goal was to assess the neuropsychological abilities of children with ASD in northern Finland and Egypt and to examine the effect of age and intelligence quotient (IQ) on these abilities.

**Design:**

Selected verbal and non-verbal subtests of the neuropsychological assessment NEPSY were used to examine 88 children with ASD in northern Finland (n=54, age M=11.2, IQ M=117.1) and Egypt (n=34, age M=8.4, IQ M=96.6).

**Results:**

Finnish ASD children scored significantly higher than their Egyptian counterparts on the verbal NEPSY subtests Comprehension of Instructions (p<0.001), Comprehension of Sentence Structure (p<0.01), Narrative Memory (p<0.001) and Verbal Fluency (p<0.05) and on the non-verbal NEPSY subtest Design Fluency (p<0.01). Finnish and Egyptian ASD children did not differ on the subtests Memory for Faces, Object Recognition and Object Memory. In addition, we found that age and verbal IQ can have significant influence on neuropsychological performance.

**Conclusions:**

Our results suggest a possible cultural impact on verbal and visuomotor fluency. However, the ability to recognize and memorize objects and the disability to remember faces appear to be typical for ASD and culturally independent.

Autism spectrum disorder (ASD) is a lifelong developmental disorder characterized by impairments in social and communication skills as well as by a restricted, repetitive, stereotyped pattern of behaviour (1). Children with ASD may present a myriad of neuropsychological features that include deficits in attention and executive function, memory and learning, language, sensorimotor function, visuospatial processing and social perception. Most of the previous studies investigating the neuropsychological functioning of children with ASD have only analysed some selected abilities such as language, attention or executive functions. More comprehensive assessment of the neuropsychological profile of children with ASD has been the focus of recent research and a number of studies have been reported (2–4). These have proved to be pivotal for our understanding of the strengths and weaknesses of the cognitive abilities of ASD patients. However, whereas most of the published evidence in ASD comes from single centres, studying ASD across countries is essential in identifying possible different phenotypes of ASD and in turn designing culturally appropriate assessment tools and treatment plans. In addition, evidence of similar neurocognitive profiles in different cultures might strengthen the notion of an innate typical profile in ASD. Previous research implemented to compare ASD in different countries or ethnicities has so far examined the prevalence rate of ASD (5–7), expression of autistic traits (8), symptom expression (9), challenging behaviours (10), comorbidity (11), parents’ treatment decisions (12) and perceptual style (13). However, there is a paucity of literature on the differences in the neuropsychological features of children with ASD across countries.

The developmental neuropsychological assessment NEPSY is one of the comprehensive tools designed for the assessment of this area of development in children (14). It was standardized and validated originally in Finland, then in the United States and some other countries. Even though both Finland and the United States are Western countries and the standardization samples in both versions were comparable (14, 15), scaled score equivalents of raw scores for NEPSY subtests differed between the Finnish and US populations. With same raw score, a child achieved a lower standard score in the Finnish version (15) than in the US version of NEPSY (14). This finding highlights the importance of considering the potential effect of cultural differences while employing neuropsychological measures in typically developing children as well as those with neuropsychological impairments such as ASD.

The purpose of this study was to assess the verbal and non-verbal neuropsychological abilities of children with ASD in Finland and Egypt. We also aimed to examine the effect of age and intelligence quotient (IQ) on these abilities.

## Methods

### Study population

Eighty-eight children with ASD in Finland (n=54) and Egypt (n=34) participated in the study (Table I). Finnish children with ASD were recruited from 2 different, partially overlapping studies in northern Finland: (a) an epidemiological study conducted in 2000–2003 (16) and (b) a genetic clinical outpatient study in 2003 (17). Egyptian children with ASD were recruited from the Child Psychiatry Outpatient Clinic of the Abbassia Mental Hospital – a tertiary referral public hospital (n=13) and from the private child psychiatric clinic of one of the authors (MO) in Cairo (n=21), over the 2 years 2007 and 2008. ASD diagnoses were based on the criteria of the International Statistical Classification of Diseases and Related Health Problems, 10th revision (1), and confirmed with the Autism Diagnostic Interview – Revised (ADI-R) (18) and Autism Diagnostic Observation Schedule (19). Children with ASD from both countries had a full scale IQ (FSIQ) higher than 70 on the Wechsler Intelligence Scale for Children – Third Revision (WISC-III) (20, 21). Severe developmental disorders (e.g. specific language impairment, epilepsy and fragile X syndrome) were used as exclusion criteria. In addition, the severity of ASD symptoms of children from both countries was assessed using the Social Responsiveness Scale (SRS) (22, 23).

**Table I:** Participants’ demographic information

	Finnish ASD children (n=54)	Egyptian ASD children (n=34)	Group comparison
Gender (boys/girls)	42/12	31/3	
Age, M (SD)	11.2 (1.7)	8.4 (2.4)	Finnish>Egyptian***
FSIQ, M (SD)	117.1 (16.2)	96.6 (19.1)	Finnish>Egyptian***
VIQ, M (SD)	126.3 (18.8)	101.1 (20.2)	Finnish>Egyptian***
PIQ, M (SD)	104.9 (14.6)	93.1 (18.5)	Finnish>Egyptian**
SRS, M (SD)	83.6 (27.3)	82.1 (21.0)	No differences
Number of siblings, M (SD)	2.5 (1.9)	1.5 (0.8)	Finnish>Egyptian**

Independent t-test. p-values:

* p<0.05

** p<0.01

*** p<0.001.

ASD, autism spectrum disorder; FSIQ, full scale IQ; VIQ, verbal IQ; PIQ, performance IQ; SRS, Social Responsiveness Scale.

### Measures

#### Wechsler Intelligent Scale for Children – Third Revision

The WISC-III is a test that assesses intelligence and cognitive abilities in children. It consists of 2 domains: the Verbal Domain (Information, Comprehension, Arithmetic, Similarities, Vocabularies and Digit Span) and the Performance Domain (Picture Completion, Picture Arrangement, Block Design, Object Assembly, Coding and Mazes) (20, 21). The Arabic version of the WISC-III employed in this study was adapted in Egypt and has Egyptian normative data (21).

#### Autism Diagnostic Interview – Revised

This is a comprehensive interview consisting of 93 questions conducted with the parent. It covers 3 areas: communication, reciprocal social interaction and stereotyped and repetitive interests/activities, which are the areas of impairment in autism (18).

#### Autism Diagnostic Observation Schedule

This is an interactive play- and conversation-oriented semi-structured observation and interview. The schedule aims to observe children’ communicative skills and explore their understanding of their and other persons’ emotions. It consists of 4 modules where the module selection is based on the verbal abilities of the child (19).

#### Social Responsiveness Scale

The SRS is a 65-item questionnaire filled out by one of the parents concerning the child. It has 5 subscales to assess different aspects of social responsiveness. These subscales are as follows: social awareness, social cognition, social communication, social motivation and autistic mannerisms (22).

#### Neuropsychological assessment

The NEPSY is a neuropsychological assessment tool administered to 3-to-12-year-old children and used to investigate children's neurocognitive development in 5 neuropsychological domains (i.e. attention and executive functions, language, sensorimotor functions, visuospatial processing and memory and learning) via 30 subtests in the Finnish version and 27 subtests in the US version (14, 15), respectively. The NEPSY subtests of Object Recognition, Object Memory and Comprehension of Sentence Structure are not included in the US NEPSY version. The psychometric properties of NEPSY are satisfactory (15). Higher scores reflect better performance. The first version of NEPSY was used in this study, as NESPY-II was not yet published at the time of data collection.

In this study, we selected the following NEPSY subtests: Memory for Faces, Object Recognition, Object Memory and Design Fluency for assessing non-verbal neuropsychological abilities. For assessing verbal neuropsychological abilities, we selected the subtests Comprehension of Instructions, Comprehension of Sentence Structure, Narrative Memory and Verbal Fluency. All subtests have verbal instructions and most of them include some kind of initial training to reduce the novelty effect and to ensure that the participant understands the task.

The Memory for Faces subtest assessing visual short term memory includes 16 cards of black and white face photos, which participants are afterwards asked to identify from 16 different sets of 3 photos. Each set has 1 photo from the cards previously seen and 2 new photos. Participants are asked to identify photos immediately and again after a 30-minute delay. The Object Recognition subtest assessing visual recognition and perception includes 13 different black and white photos of non-facial objects with 3 different levels of pixel sharpness (fuzzy, partly fuzzy and sharp). The participant is asked to identify objects first from the fuzziest photos, second from the partly fuzzy photos and last from the sharp photos. Afterwards participants are shown 4 sets of 13 sharp photos each, which form the Object Memory subtest assessing short-term memory, and participants are asked to identify an object seen earlier from 4 choices. The Design Fluency subtest evaluates non-verbal fluency by asking the participant to draw different figures by connecting dots with lines within a 2-minute time frame. The Comprehension of Instructions subtest evaluates the participant's ability to understand and respond quickly to 28 verbal instructions of increasing complexity. The Comprehension of Sentence Structure subtest evaluates participants’ ability to understand grammar with 21 sentences. The Narrative Memory subtest assessing auditive working memory evaluates the participant's ability to repeat a story he or she just heard. The Verbal Fluency subtest examines the participant's ability to produce words that belong to certain semantic (e.g. animals, food or drinks) and phonemic (e.g. words starting with the letters *s* or *f*) categories within 1 minute.

For the purpose of the current study, the employed verbal NEPSY subtests were translated from English into Arabic. In addition, the NEPSY subtest of Comprehension of Sentence Structure was translated from the Finnish version of NEPSY into Arabic. A process of repeated translation and back-translation took place to reach a congruent Arabic version. Afterwards the translated items were further assessed by a bilingual (Arabic and English), highly qualified, clinical psychologist (author GR). More specifically, in the Narrative Memory subtest, the Arabic version of the story has the same sequence of events, same number of sentences and same total number of words as in the US NEPSY version. As for choosing suitable Arabic letters for the phonemic part of Verbal Fluency subtest, the letters *s* (*seen*) and *f* (*faa*) were found to be appropriate regarding the frequency of words that start with these letters as well as the fact that these 2 letters are not commonly attached to words as prefixes.

### Ethical consideration

The study was approved by the Ethics Committee of the Northern Ostrobothnia Hospital District, Finland, and by the Ethical Committee of the Rights of Human Subjects in Scientific Research of the General Secretariat of Mental Health in Cairo, Egypt. The procedure was explained to all participants’ parents and written consents were obtained.

### Statistical analysis

The statistical analyses were carried out using SPSS 19.0 statistical software program for Macintosh. An analysis with an independent t-test was employed to examine the differences between groups in age, FSIQ, verbal IQ (VIQ), performance IQ (PIQ), severity of ASD symptoms and family size (Table I).

Differences in NEPSY results between Finnish and Egyptian ASD children as well as the effect of age, IQ and country (Finland and Egypt) were obtained using multivariate analysis of covariance (MANCOVA). As there were significant age and IQ differences between ASD groups, MANCOVA was conducted with the NEPSY raw scores as dependent variables, country as a fixed factor and age and IQ (VIQ for the verbal NEPSY subtests and PIQ for the non-verbal NEPSY subtests) as covariates. Raw NEPSY scores were used instead of age-normed standard scores because NEPSY is standardized for Finnish and US children, but not for Egyptian children.

Pearson's correlation coefficient was computed to study the association between NEPSY subtests and the severity of ASD symptoms (SRS) in both countries.

Effect size was evaluated using the eta-squared ($\eta_{p}^{2}$) statistic. According to Cohen, $\eta_{p}^{2}$=0.01 is considered a small effect, $\eta_{p}^{2}$=0.06 a medium effect and $\eta_{p}^{2}$=0.14 a large effect (24). All tests of statistical significance are reported as two-tailed and p<0.05 was considered significant.

## Results

Children with ASD in Finland scored significantly higher than those in Egypt in all employed verbal NEPSY subtests and in the non-verbal NEPSY subtest Design Fluency. There were no differences found between Finnish and Egyptian ASD children in the non-verbal subtests Memory for Faces, Object Recognition and Object Memory (Table II). The children's performance improved with age in all verbal NEPSY subtests and in all non-verbal NEPSY subtests other than Object Memory (Table III).

**Table II:** Differences between Finnish and Egyptian children with ASD on NEPSY subtests using MANCOVA to control for age and IQ

NEPSY tasks	Finland, M (SD)	Egypt, M (SD)	F	p-value	Partial eta^2^
Non-verbal subtests:					
Memory for Faces	25.3 (4.3)	20.7 (4.6)	3.2	0.08	0.04
Object Recognition	20.6 (4.8)	14.4 (5.7)	0.0	0.95	0.00
Object Memory	10.7 (2.1)	8.3 (2.2)	0.4	0.55	0.01
Design Fluency	26.0 (7.8)	9.6 (5.9)	8.4	<**0.01**	0.09
Verbal subtests:					
Comprehension of Instruction	25.1 (3.1)	16.0 (3.9)	23.9	<**0.001**	0.24
Comprehension of Sentence Structure	17.0 (3.4)	13.5 (3.4)	7.3	<**0.01**	0.09
Narrative Memory	25.2 (4.7)	10.8 (9.1)	29.1	<**0.001**	0.27
Verbal Fluency	48.4 (16.7)	19.4 (11.8)	6.8	<**0.05**	0.09

Significant differences appear in boldface. NEPSY, a neuropsychological assessment; MANCOVA, multivariate analysis of covariance.

**Table III:** Effect of age, IQ and interactions between country and IQ on NEPSY performance of ASD children using MANCOVA to control for age and IQ

	Effect of age		Effect of IQ		Interaction between country and IQ	
			
	F	Partial eta^2^	F	Partial eta^2^	F	Partial eta^2^
Non-verbal subtests:						
Memory for Faces	11.9***	0.13	0.0	00	2.0	0.03
Object Recognition	11.5***	0.16	2.7	0.05	0.0	0.00
Object Memory	1.0	0.02	1.0	0.02	1.2	0.02
Design Fluency	13.8***	0.15	3.9	0.05	2.8	0.04
Verbal subtests:						
Comprehension of Instruction	20.9***	0.21	8.2**	0.09	12.0***	0.13
Comprehension of Sentence Structure	9.3*	0.11	4.3*	0.05	6.1*	0.07
Narrative Memory	16.3***	0.17	23.7***	0.23	19.7***	0.20
Verbal Fluency	7.8*	0.10	3.7	0.05	2.8	0.04

Performance IQ was used for the analysis of performance in non-verbal NEPSY subtests; verbal IQ was used for the analysis of performance in verbal subtests; p-values:

* p<0.05

** p<0.01

*** p<0.001.

**Fig. 1:**
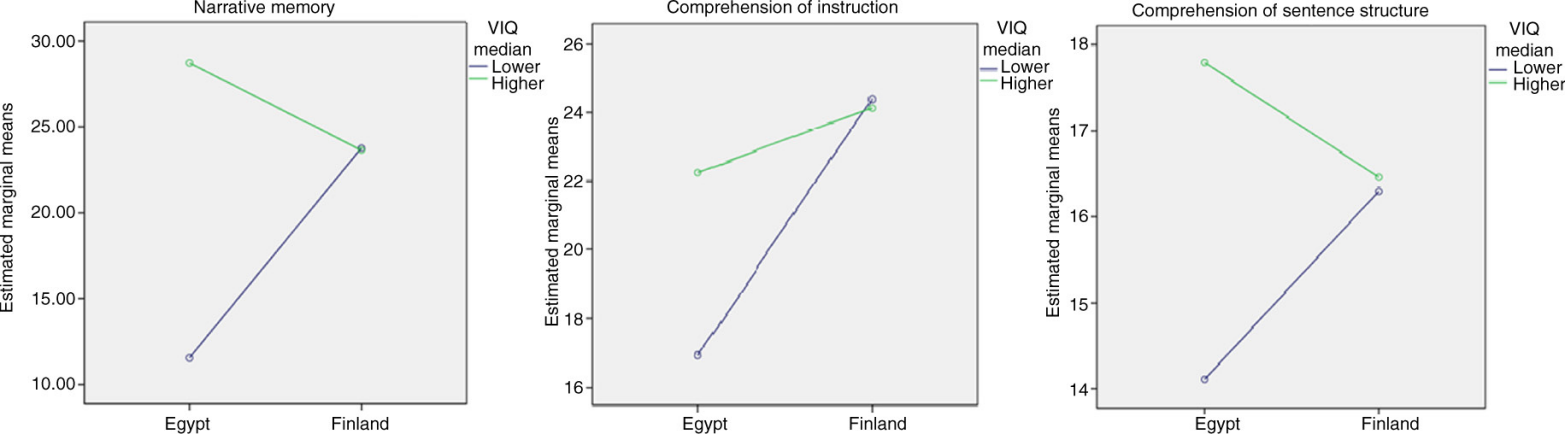
Interaction of country and verbal IQ (VIQ) in NEPSY subtests among ASD children.

There was a significant effect of VIQ on all verbal NEPSY subtests except Verbal Fluency among ASD children, whereas there was no significant effect of PIQ on any of the non-verbal subtests (Table III). Specifically, a higher VIQ was associated with a higher score on the Comprehension of Instruction, Comprehension of Sentence Structure and Narrative Memory subtests among Egyptian children with ASD (Table III and Fig. 1).

To avoid potential bias that may originate from the significant age differences between the study groups, we repeated MANCOVA analysis including only children 8–10 years old (Finnish ASD, n=23, age M (SD)=9.6 (0.9); Egyptian ASD, n=12, age M (SD)=9.5 (0.7), p=0.064) and controlling for IQ. The findings were in accordance with the findings from the whole study sample, except regarding the subtest Comprehension of Sentence Structure, which was no longer statistically significant. Specifically, the Finnish ASD children scored significantly higher than those in Egypt in all employed verbal NEPSY subtests (Comprehension of Instruction, F=10.1, p=0.004, ηp^2^=0.29; Narrative Memory, F=6.7, p=0.016, ηp^2^=0.21; Verbal Fluency, F=4.4, p=0.046, ηp^2^=0.15) apart from Comprehension of Sentence Structure (F=2.8, p=0.107, ηp^2^=0.10) and in the non-verbal NEPSY subtest Design Fluency (F=22.9, p=0.000, ηp^2^=0.54). There were no differences found between the Finnish and Egyptian ASD children in the non-verbal subtests Memory for Faces (F=3.9, p=0.061, ηp^2^=0.17), Object Recognition (F=0.1, p=0.705, ηp^2^=0.00) and Object Memory (F=0.0, p=0.902, ηp^2^=0.00).

### Association between NEPSY scores and severity of autism

The Finnish and Egyptian ASD children did not differ significantly in SRS scores (see Table I). However, there was a significant inverse correlation between total SRS score (i.e. severity of ASD symptoms) and performance on the NEPSY subtest Comprehension of Instruction (r=−0.40, p<0.05) among the Egyptian ASD children. There were no significant correlations between NEPSY subtests and SRS among the Finnish children with ASD.

## Discussion

There is a gap in the literature regarding the differences in neuropsychological performance of ASD children across countries. The current study aimed to compare the neuropsychological abilities of children with ASD in Finland (a Western culture) and those in Egypt (an Eastern culture), as well as to evaluate various factors that may affect the neuropsychological performance of ASD children. Overall, the Egyptian children scored lower than the Finnish children on the employed neuropsychological subtests.

The Finnish children with ASD were more fluent both verbally and non-verbally when compared to their Egyptian counterparts. Previous studies have demonstrated the effect of culture on verbal and non-verbal fluencies in healthy individuals (25–27). Khalil (2010) showed that healthy Saudi Arabian adults scored lower than non-Saudi Arabian adults in the fluency of semantic categories (animals) (25). The author attributed this difference to the fact that Saudi Arabians are less likely to be exposed (e.g. via TV or books) to wild animals and hence were producing mostly the names of domestic animals (25). However, this possibility does not apply to our results, as the proportions of domestic animals (53.4%) and wild animals in our Egyptian cohort were not very different. Moreover, in support of the effect of culture on semantic fluency, significant difficulties in verbal fluency were found among a cohort of Mayan adults (Native Americans of southern Mexico), explained partly by low level of education (1–4 years) (26). In regard to non-verbal fluency tasks, Mulenga and colleagues found that Zambian children performed poorer than US children in the NEPSY domain of attention and executive function, which includes the Design Fluency subtest (27). This finding might be a result of the culture effect on time-limited tasks (27, 28). In our study, we did not employ time-limited tasks other than verbal and non-verbal fluency tests; thus it is difficult to determine whether the limited time had a cultural effect on our study population. Additionally, individuals with high anxiety traits are expected to perform worse on neuropsychological tasks involving inhibition, shifting and working memory as in fluency subtests (29, 30). One recent study emphasized the passive effect of anxiety on performance in design fluency tasks (31), which raises the question of whether the anxiety trait is different from one country to another.

Regarding the performance of children with ASD in verbal tasks, we found that the Egyptian children did not perform as well as their Finnish counterparts. It is possible that the phonetically regular and transparent orthography of the Finnish language may facilitate the comprehension process in ASD children (32). However, the differences on the Comprehension of Instruction subtest should be tentatively interpreted due to the potential bias that may originate from the age differences between our study groups, particularly because differences in Comprehension of Instruction subtest results were not significant when restricting the analyses to children between 8 and 10 years old in both groups.

The trend of lower neuropsychological abilities among the Egyptian children with ASD compared to the Finnish children with ASD may be attributed to lack of access to services (either because of cost or availability) and lack of awareness among the general Egyptian population. Moreover, lack of child psychiatric training of mental health professionals, paediatricians and primary care physicians in Egypt may lead to a delay in diagnosis and treatment of comorbid mental health problems and thus the sensitive period for learning skills may be missed (33, 34). A recent study shows that delayed psychiatric consultations among Egyptians are common in the lower and middle social classes and most of the parents sought advice first from paediatricians or traditional healers (33). Furthermore, a multidisciplinary comprehensive therapy and special education are available only for a limited number of diagnosed cases in Egypt due to financial constraints, which is not the case in Finland where these services are provided by the government for all children without any financial burden on the family.

We found that there was an insignificant difference between the Finnish and Egyptian children on the non-verbal subtests of memory for faces and objects as well as on object recognition. This finding raises another question: Are those tasks culturally independent neuropsychological abilities? Previous studies have shown that ASD children perform similarly with typically developing children in tasks requiring fact-memory or non-socially related memory, whereas socially related memory and face memory may be impaired (35, 36).

With respect to the effect of a child's age and consequently his or her years of education, our findings are consistent with other studies that reported positive relation between age and neuropsychological performance in general (26, 37) and in verbal fluency (25–27), design fluency (38), object recognition (35) and narrative memory (39) in particular. Similar to a study by Kuusikko-Gauffin and colleagues (35), we did not find an age effect on performance on object memory tasks among ASD children.

Our results regarding IQ effect on the neuropsychological performance of ASD children demonstrated the impact of VIQ on verbal neuropsychological tasks of comprehension of instruction, comprehension of sentence structure and narrative memory but not on the verbal fluency task. The absence of association between VIQ and verbal fluency tasks was also noted in another study (3). It could be that the task is time-limited and therefore more dependent on executive functions, such as cognitive flexibility, than on VIQ. In our study, there was no significant effect of PIQ on the employed non-verbal neuropsychological tasks, which may be due to generally lower PIQs than VIQs in ASD populations. There is a wide diversity of findings with respect to the effect of IQ on the neuropsychological performance of ASD children: some authors found a significant correlation (40) and others did not find any association (3).

To the best of our knowledge, this study is the first to assess the similarities and differences across countries in the neuropsychological abilities of children with ASD. Native multiprofessional ADI-R-trained teams carried out this study, in both countries, to ensure a good understanding of participants’ culture and language. However, some limitations of this study should be noted. The small number of participants may have led to the introduction of bias into the results; thus more studies with bigger sample sizes across countries are needed to fully understand the impact of culture on neuropsychological performance in ASD. Moreover, despite our effort to match the Finnish and Egyptian children, significant differences in age and IQ still existed and hence we chose to use MANCOVA in our analysis to control the age and IQ facts. However, our findings were confirmed with a smaller age-matched study sample.

## Conclusions

In summary, the results of our study suggest a possible cultural impact on verbal abilities in ASD children, whereas memory for faces, memory for objects and object recognition are likely to be culturally independent abilities that may be similarly mastered or impaired in children with ASD regardless of their country of origin. Finally, similar to reported literature, we found that age and VIQ can have significant influence on neuropsychological performance.
